# Poly[[diaqua­bis(μ_2_-isonicotinato-κ^2^ *N*:*O*)bis­(μ_3_-isonicotinato-κ^3^ *N*:*O*:*O*′)neodymium(III)disilver(I)] nitrate monohydrate]

**DOI:** 10.1107/S1600536809042342

**Published:** 2009-10-28

**Authors:** Qing-Guang Zhan, Jie-Xuan Huang, Rong-Hua Zeng

**Affiliations:** a School of Chemistry and Environment, South China Normal University, Guangzhou 510006, People’s Republic of China; b Key Laboratory of Technology on Electrochemical Energy, Storage and Power Generation in Guangdong Universities, South China Normal University, Guangzhou 510006, People’s Republic of China

## Abstract

In the title complex, {[Ag_2_Nd(C_6_H_4_NO_2_)_4_(H_2_O)_2_]NO_3_·H_2_O}_*n*_, the Nd^III^ ion is coordinated by eight O atoms from six isonicotinate ligands and two water mol­ecules in a distorted square anti­prismatic geometry. Each Ag^I^ ion is coordinated by two N atoms from two different isonicotinate ligands. The crystal structure exhibits a two-dimensional heterometallic polymeric layer. O—H⋯O hydrogen bonds involving the coordinated and uncoordinated water mol­ecules and intra­layer π–π inter­actions between the pyridine rings [centroid–centroid distances = 3.571 (2) and 3.569 (2) Å] are observed. Each layer inter­acts with two neighboring ones *via* Ag⋯O(H_2_O) contacts and inter­layer π–π inter­actions [centroid–centroid distances = 3.479 (3) to 3.530 (3) Å], leading to a three-dimensional supra­molecular network.

## Related literature

For general background to metal organic frameworks, see: Batten & Robson (1998 ▶); Min & Suh (2000 ▶). For 4*d*–4*f* heterometallic coordination frameworks, see: Cai *et al.* (2009 ▶).
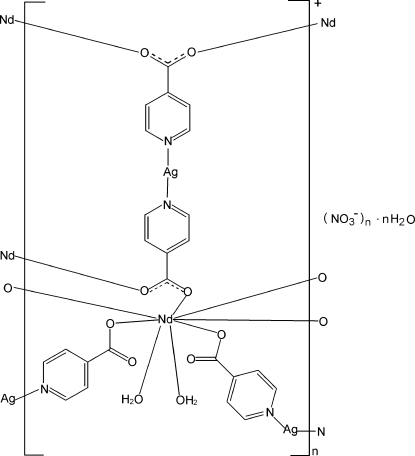


## Experimental

### 

#### Crystal data

[Ag_2_Nd(C_6_H_4_NO_2_)_4_(H_2_O)_2_]NO_3_·H_2_O
*M* *_r_* = 964.45
Monoclinic, 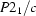

*a* = 16.9648 (19) Å
*b* = 24.793 (3) Å
*c* = 6.7770 (8) Å
β = 95.849 (1)°
*V* = 2835.7 (6) Å^3^
*Z* = 4
Mo *K*α radiation
μ = 3.25 mm^−1^
*T* = 296 K
0.23 × 0.20 × 0.18 mm

#### Data collection

Bruker APEXII CCD diffractometer
Absorption correction: multi-scan (*SADABS*; Sheldrick, 1996 ▶) *T*_min_ = 0.522, *T*_max_ = 0.592
14629 measured reflections
5092 independent reflections
4024 reflections with *I* > 2σ(*I*)
*R*_int_ = 0.043

#### Refinement

*R*[*F* ^2^ > 2σ(*F* ^2^)] = 0.032
*wR*(*F* ^2^) = 0.067
*S* = 1.01
5092 reflections
433 parameters
9 restraints
H atoms treated by a mixture of independent and constrained refinement
Δρ_max_ = 0.57 e Å^−3^
Δρ_min_ = −0.91 e Å^−3^

### 

Data collection: *APEX2* (Bruker, 2007 ▶); cell refinement: *SAINT* (Bruker, 2007 ▶); data reduction: *SAINT*; program(s) used to solve structure: *SHELXS97* (Sheldrick, 2008 ▶); program(s) used to refine structure: *SHELXL97* (Sheldrick, 2008 ▶); molecular graphics: *DIAMOND* (Brandenburg, 1999 ▶); software used to prepare material for publication: *SHELXL97*.

## Supplementary Material

Crystal structure: contains datablocks I, global. DOI: 10.1107/S1600536809042342/hy2237sup1.cif

Structure factors: contains datablocks I. DOI: 10.1107/S1600536809042342/hy2237Isup2.hkl

Additional supplementary materials:  crystallographic information; 3D view; checkCIF report

## Figures and Tables

**Table 1:** Selected bond lengths (Å)

Nd1—O1^i^	2.381 (3)
Nd1—O2	2.502 (3)
Nd1—O3^ii^	2.426 (3)
Nd1—O4^iii^	2.432 (3)
Nd1—O5	2.416 (3)
Nd1—O7	2.406 (3)
Nd1—O2*W*	2.492 (4)
Nd1—O3*W*	2.564 (3)
Ag1—N1	2.155 (4)
Ag1—N2	2.155 (4)
Ag1⋯O1*W*^i^	2.888 (4)
Ag1⋯O10	2.771 (5)
Ag2—N3^iv^	2.200 (4)
Ag2—N4	2.184 (4)
Ag2⋯O3*W*^v^	2.741 (4)
Ag2⋯O9^vi^	2.950 (5)

Symmetry codes: (i) 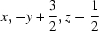; (ii) 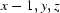; (iii) 
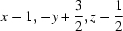
; (iv) 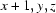; (v) 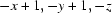; (vi) 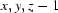.

**Table 2:** Hydrogen-bond geometry (Å, °)

*D*—H⋯*A*	*D*—H	H⋯*A*	*D*⋯*A*	*D*—H⋯*A*
O1*W*—H1*A*⋯O10^vii^	0.85 (3)	1.96 (2)	2.796 (8)	167 (7)
O1*W*—H1*B*⋯O9	0.85 (4)	2.12 (5)	2.945 (8)	164 (6)
O2*W*—H2*A*⋯O6	0.85 (4)	2.06 (3)	2.799 (5)	145 (5)
O2*W*—H2*B*⋯O4^iii^	0.84 (4)	2.21 (4)	3.033 (5)	166 (5)
O3*W*—H3*A*⋯O8	0.85 (3)	1.87 (2)	2.653 (5)	153 (4)
O3*W*—H3*B*⋯O6^vii^	0.85 (3)	2.11 (3)	2.951 (5)	174 (5)

Symmetry codes: (iii) 
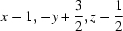
; (vii) 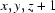.

**Fig. 1.**
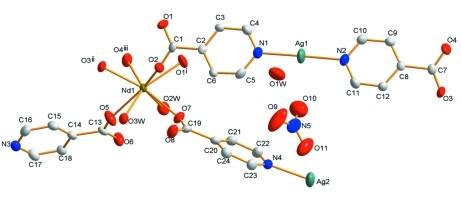
The asymmetric unit of the title compound. Displacement ellipsoids are drawn at the 30% probability level. H atoms have been omitted for clarity. [Symmetry codes: (i) x, 3/2-y, -1/2+z; (ii) -1+x, y, z; (iii) -1+x, 3/2-y, -1/2+z.]

**Fig. 2.**
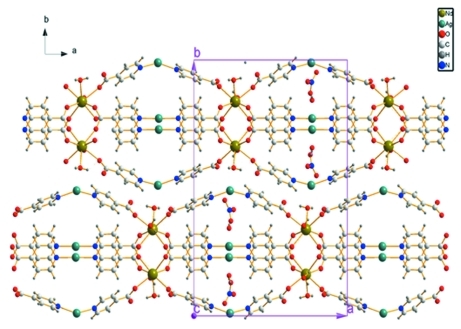
A view of the layered networks in the title compound.

## supplementary crystallographic information

### Comment

In recent years, assembly processes directed by metal–ligand ligation have been extensively utilized to construct metal organic frameworks with novel topologies and potentially interesting functions in magnetism, photoluminescence, sorption, catalysis (Batten & Robson, 1998; Min & Suh, 2000). However, metal-directed assembly of 4d–4f heterometallic coordination frameworks with fascinating topological networks and potential applications have been few reported (Cai *et al.*, 2009). We utilized isonicotinate as multifunctional ligand with O and N atoms on opposite sites. Here, a new metal-directed assembly of 4d–4f coordination polymer, which was synthesized under hydrothermal conditions, is reported.

The asymmetric unit of the title complex contains one Nd^III^ ion, two Ag^I^ ions, four crystallographically unique isonicotinate ligands, one nitrate anion, two coordinated water molecules and one uncoordinated water molecule (Fig. 1). The Nd^III^ ion is in a distorted square antiprismatic geometry, defined by eight O atoms from six isonicotinate ligands and two water molecules. The Nd—O bond distances and O—Nd—O bond angles range from 2.381 (3) to 2.564 (3) Å and 71.79 (11) to 145.83 (12)°, respectively (Table 1). The Ag^I^ ion exhibits an approximatly linear or bow-like configuration, being coordinated by two N atoms from two different isonicotinate ligands. The isonicotinate ligands link Nd and Ag metal centers, forming a layer in the (010) plane, which are stabilized by O—H···O hydrogen bonds involving the coordinated and uncoordinated water molecules (Table 2) and intralayer π–π stacking interactions between the pyridine rings, with a centroid–centroid distances of 3.571 (2) and 3.569 (2) Å. These layers are further connected by Ag···O(H_2_O) contacts and interlayer π–π stacking interactions [centroid–centroid distances = 3.479 (3) to 3.530 (3) Å] between the pyridine rings of two adjacent layers, assembling a three-dimensional supramolecular architecture (Fig. 2).

### Experimental

A mixture of Nd_2_O_3_ (0.183 g, 0.5 mmol), AgNO_3_ (0.170 g, 1 mmol), isonicotinic acid (0.135 g, 1.5 mmol), water (10 ml) in the presence of HNO_3_(0.024 g, 0.385 mmol) was stirred vigorously for 20 min and then sealed in a Teflon-lined stainless-steel autoclave (20 ml capacity). The autoclave was heated and maintained at 443 K for 3 d, and then cooled to room temperature at 5 K h^-1^.  The colorless block crystals of the title compound were obtained.

### Refinement

Water H atoms were tentatively located in difference Fourier maps and refined with distance restraints of O—H = 0.85 (1) Å and H···H = 1.35 (1) Å, and with *U*_iso_(H) = 1.5*U*_eq_(O). H atoms attached to C atoms were placed at calculated positions and treated as riding on their parent atoms, with C—H = 0.93 Å and with *U*_iso_(H) = 1.2*U*_eq_(C).

### Crystal data

**Table:**

[Ag_2_Nd(C_6_H_4_NO_2_)_4_(H_2_O)_2_]NO_3_·H_2_O	*F*(000) = 1868
*M**_r_* = 964.45	*D*_x_ = 2.259 Mg m^−^^3^
Monoclinic, *P*2_1_/*c*	Mo *K*α radiation, λ = 0.71073 Å
Hall symbol: -P 2ybc	Cell parameters from 3327 reflections
*a* = 16.9648 (19) Å	θ = 2.4–25.9°
*b* = 24.793 (3) Å	µ = 3.25 mm^−^^1^
*c* = 6.7770 (8) Å	*T* = 296 K
β = 95.849 (1)°	Block, colorless
*V* = 2835.7 (6) Å^3^	0.23 × 0.20 × 0.18 mm
*Z* = 4	

### Data collection

**Table:**

Bruker APEXII CCD diffractometer	5092 independent reflections
Radiation source: fine-focus sealed tube	4024 reflections with *I* > 2σ(*I*)
graphite	*R*_int_ = 0.043
φ and ω scans	θ_max_ = 25.2°, θ_min_ = 1.6°
Absorption correction: multi-scan (*SADABS*; Sheldrick, 1996)	*h* = −17→20
*T*_min_ = 0.522, *T*_max_ = 0.592	*k* = −29→29
14629 measured reflections	*l* = −8→5

### Refinement

**Table:**

Refinement on *F*^2^	Primary atom site location: structure-invariant direct methods
Least-squares matrix: full	Secondary atom site location: difference Fourier map
*R*[*F*^2^ > 2σ(*F*^2^)] = 0.032	Hydrogen site location: inferred from neighbouring sites
*wR*(*F*^2^) = 0.067	H atoms treated by a mixture of independent and constrained refinement
*S* = 1.01	*w* = 1/[σ^2^(*F*_o_^2^) + (0.0268*P*)^2^] where *P* = (*F*_o_^2^ + 2*F*_c_^2^)/3
5092 reflections	(Δ/σ)_max_ = 0.001
433 parameters	Δρ_max_ = 0.57 e Å^−^^3^
9 restraints	Δρ_min_ = −0.91 e Å^−^^3^

### Fractional atomic coordinates and isotropic or equivalent isotropic displacement parameters (Å^2^)

**Table:**

	*x*	*y*	*z*	*U*_iso_*/*U*_eq_	
Nd1	0.269306 (14)	0.662399 (10)	0.11749 (4)	0.01959 (8)	
Ag1	0.77067 (2)	0.728604 (19)	0.49039 (7)	0.04705 (14)	
Ag2	0.76495 (3)	0.510435 (19)	−0.09689 (7)	0.05046 (14)	
O7	0.38451 (19)	0.60656 (13)	0.1017 (5)	0.0338 (9)	
O2	0.35364 (18)	0.68568 (12)	0.4305 (5)	0.0289 (8)	
O5	0.1656 (2)	0.60334 (15)	−0.0262 (5)	0.0456 (10)	
C3	0.5223 (3)	0.77468 (17)	0.5011 (7)	0.0242 (11)	
H3	0.4958	0.8074	0.5054	0.029*	
C1	0.3912 (3)	0.72711 (19)	0.4857 (7)	0.0229 (11)	
C2	0.4801 (3)	0.72666 (17)	0.4924 (6)	0.0197 (10)	
N2	0.8974 (2)	0.72776 (16)	0.4834 (6)	0.0341 (10)	
N1	0.6441 (2)	0.72777 (16)	0.4996 (6)	0.0327 (10)	
C6	0.5224 (3)	0.67929 (18)	0.4894 (7)	0.0246 (11)	
H6	0.4963	0.6462	0.4835	0.030*	
C10	0.9388 (3)	0.77404 (19)	0.4767 (7)	0.0308 (12)	
H10	0.9122	0.8067	0.4841	0.037*	
C9	1.0183 (3)	0.77478 (18)	0.4595 (7)	0.0275 (12)	
H9	1.0448	0.8076	0.4563	0.033*	
C20	0.5069 (3)	0.56100 (17)	0.1414 (7)	0.0258 (11)	
C19	0.4289 (3)	0.57592 (18)	0.2152 (8)	0.0259 (11)	
O8	0.4127 (2)	0.55701 (14)	0.3746 (5)	0.0371 (9)	
N4	0.6493 (2)	0.52916 (15)	0.0010 (6)	0.0316 (10)	
C22	0.5972 (3)	0.56051 (19)	−0.1083 (8)	0.0337 (13)	
H22	0.6094	0.5719	−0.2323	0.040*	
C23	0.6300 (3)	0.5135 (2)	0.1793 (8)	0.0341 (13)	
H23	0.6649	0.4912	0.2561	0.041*	
C5	0.6032 (3)	0.6814 (2)	0.4951 (8)	0.0334 (13)	
H5	0.6312	0.6491	0.4959	0.040*	
C8	1.0594 (3)	0.72707 (18)	0.4469 (6)	0.0218 (11)	
C12	1.0174 (3)	0.67961 (19)	0.4565 (7)	0.0279 (12)	
H12	1.0430	0.6466	0.4493	0.033*	
C14	0.0414 (3)	0.57066 (17)	−0.1642 (7)	0.0242 (11)	
C15	0.0089 (3)	0.57587 (18)	0.0129 (7)	0.0308 (12)	
H15	0.0389	0.5904	0.1228	0.037*	
C13	0.1266 (3)	0.58696 (18)	−0.1849 (8)	0.0277 (12)	
C18	−0.0059 (3)	0.54950 (18)	−0.3233 (7)	0.0291 (12)	
H18	0.0130	0.5466	−0.4469	0.035*	
O6	0.1513 (2)	0.58320 (14)	−0.3490 (5)	0.0413 (10)	
C24	0.5615 (3)	0.52881 (19)	0.2536 (8)	0.0319 (12)	
H24	0.5514	0.5176	0.3796	0.038*	
C21	0.5267 (3)	0.57646 (18)	−0.0447 (8)	0.0299 (12)	
H21	0.4919	0.5977	−0.1263	0.036*	
C16	−0.0679 (3)	0.55962 (19)	0.0274 (8)	0.0348 (13)	
H16	−0.0895	0.5645	0.1469	0.042*	
C17	−0.0811 (3)	0.53274 (19)	−0.2966 (8)	0.0341 (13)	
H17	−0.1117	0.5176	−0.4043	0.041*	
C4	0.6027 (3)	0.77385 (19)	0.5035 (7)	0.0316 (12)	
H4	0.6299	0.8064	0.5080	0.038*	
C11	0.9378 (3)	0.6811 (2)	0.4766 (7)	0.0323 (12)	
H11	0.9107	0.6487	0.4859	0.039*	
O1	0.3610 (2)	0.76974 (13)	0.5428 (5)	0.0410 (10)	
C7	1.1467 (3)	0.72688 (19)	0.4130 (7)	0.0249 (11)	
O3	1.17260 (19)	0.68473 (13)	0.3448 (5)	0.0321 (8)	
O4	1.1839 (2)	0.76939 (14)	0.4499 (6)	0.0419 (10)	
O3W	0.2587 (2)	0.57863 (14)	0.3352 (5)	0.0308 (8)	
H3B	0.2294 (19)	0.578 (2)	0.429 (4)	0.046*	
H3A	0.3050 (10)	0.571 (2)	0.387 (6)	0.046*	
O2W	0.2838 (2)	0.64789 (16)	−0.2408 (6)	0.0436 (10)	
H2A	0.257 (2)	0.6245 (15)	−0.310 (7)	0.065*	
H2B	0.251 (2)	0.6703 (17)	−0.205 (8)	0.065*	
N5	0.7673 (3)	0.5865 (2)	0.3990 (10)	0.0570 (15)	
O11	0.7991 (3)	0.5483 (2)	0.3274 (7)	0.0789 (15)	
O9	0.7419 (3)	0.5845 (2)	0.5614 (7)	0.094 (2)	
O10	0.7590 (3)	0.6295 (2)	0.3006 (8)	0.0892 (17)	
N3	−0.1129 (2)	0.53684 (16)	−0.1258 (7)	0.0326 (10)	
O1W	0.7704 (3)	0.65730 (16)	0.9046 (8)	0.0724 (14)	
H1A	0.769 (5)	0.644 (2)	1.019 (4)	0.109*	
H1B	0.763 (5)	0.6313 (17)	0.824 (7)	0.109*	

### Atomic displacement parameters (Å^2^)

**Table:**

	*U*^11^	*U*^22^	*U*^33^	*U*^12^	*U*^13^	*U*^23^
Nd1	0.01421 (13)	0.02069 (13)	0.02424 (15)	−0.00081 (11)	0.00374 (10)	0.00027 (11)
Ag1	0.0152 (2)	0.0632 (3)	0.0635 (3)	0.0000 (2)	0.00745 (19)	−0.0005 (2)
Ag2	0.0231 (2)	0.0605 (3)	0.0700 (4)	0.0006 (2)	0.0150 (2)	0.0039 (2)
O7	0.028 (2)	0.037 (2)	0.037 (2)	0.0145 (16)	0.0071 (16)	0.0030 (16)
O2	0.0205 (18)	0.0311 (18)	0.035 (2)	−0.0041 (15)	0.0004 (15)	−0.0064 (15)
O5	0.041 (2)	0.062 (2)	0.033 (2)	−0.028 (2)	0.0013 (18)	−0.0006 (19)
C3	0.024 (3)	0.019 (2)	0.030 (3)	0.001 (2)	0.002 (2)	−0.001 (2)
C1	0.017 (3)	0.032 (3)	0.021 (3)	0.003 (2)	0.006 (2)	0.003 (2)
C2	0.017 (2)	0.027 (2)	0.015 (3)	0.002 (2)	−0.0024 (19)	−0.0007 (19)
N2	0.021 (2)	0.040 (3)	0.042 (3)	0.005 (2)	0.0059 (19)	0.000 (2)
N1	0.022 (2)	0.036 (3)	0.040 (3)	0.003 (2)	0.0046 (19)	0.002 (2)
C6	0.020 (3)	0.025 (3)	0.029 (3)	−0.003 (2)	0.004 (2)	−0.003 (2)
C10	0.025 (3)	0.028 (3)	0.039 (3)	0.006 (2)	0.000 (2)	−0.001 (2)
C9	0.027 (3)	0.023 (3)	0.033 (3)	−0.002 (2)	0.003 (2)	−0.002 (2)
C20	0.025 (3)	0.018 (2)	0.034 (3)	0.000 (2)	0.001 (2)	−0.002 (2)
C19	0.024 (3)	0.020 (2)	0.034 (3)	0.003 (2)	0.003 (2)	−0.005 (2)
O8	0.035 (2)	0.046 (2)	0.032 (2)	0.0109 (18)	0.0120 (17)	0.0124 (18)
N4	0.024 (2)	0.028 (2)	0.044 (3)	−0.0003 (19)	0.007 (2)	−0.004 (2)
C22	0.031 (3)	0.030 (3)	0.042 (4)	0.003 (2)	0.010 (2)	0.005 (2)
C23	0.023 (3)	0.034 (3)	0.045 (4)	0.005 (2)	−0.001 (2)	0.002 (3)
C5	0.024 (3)	0.031 (3)	0.044 (4)	0.012 (2)	0.003 (2)	0.000 (2)
C8	0.019 (3)	0.029 (3)	0.017 (3)	−0.001 (2)	0.0016 (19)	−0.003 (2)
C12	0.022 (3)	0.025 (3)	0.037 (3)	0.002 (2)	0.003 (2)	−0.001 (2)
C14	0.024 (3)	0.019 (2)	0.029 (3)	−0.004 (2)	0.000 (2)	0.000 (2)
C15	0.035 (3)	0.028 (3)	0.029 (3)	−0.005 (2)	0.003 (2)	−0.006 (2)
C13	0.028 (3)	0.023 (3)	0.031 (3)	−0.008 (2)	0.000 (2)	−0.002 (2)
C18	0.029 (3)	0.030 (3)	0.029 (3)	−0.003 (2)	0.006 (2)	−0.002 (2)
O6	0.035 (2)	0.057 (3)	0.033 (2)	−0.0149 (19)	0.0110 (17)	−0.0100 (18)
C24	0.029 (3)	0.033 (3)	0.034 (3)	−0.001 (2)	0.001 (2)	0.001 (2)
C21	0.032 (3)	0.025 (3)	0.033 (3)	0.002 (2)	0.003 (2)	0.002 (2)
C16	0.032 (3)	0.038 (3)	0.035 (3)	0.006 (3)	0.012 (2)	−0.001 (2)
C17	0.030 (3)	0.035 (3)	0.036 (3)	−0.009 (2)	−0.005 (3)	0.003 (2)
C4	0.027 (3)	0.028 (3)	0.040 (3)	−0.007 (2)	0.002 (2)	0.000 (2)
C11	0.026 (3)	0.032 (3)	0.040 (3)	−0.006 (2)	0.004 (2)	0.004 (2)
O1	0.022 (2)	0.034 (2)	0.067 (3)	0.0073 (16)	0.0020 (17)	−0.0206 (19)
C7	0.020 (3)	0.032 (3)	0.022 (3)	0.001 (2)	0.000 (2)	0.003 (2)
O3	0.0211 (19)	0.037 (2)	0.040 (2)	0.0038 (16)	0.0118 (15)	−0.0043 (17)
O4	0.022 (2)	0.043 (2)	0.061 (3)	−0.0105 (17)	0.0040 (17)	−0.0183 (19)
O3W	0.026 (2)	0.034 (2)	0.033 (2)	0.0006 (17)	0.0093 (15)	0.0029 (16)
O2W	0.043 (2)	0.058 (3)	0.030 (2)	−0.010 (2)	0.0055 (18)	−0.0101 (18)
N5	0.033 (3)	0.068 (4)	0.066 (5)	0.011 (3)	−0.011 (3)	−0.016 (4)
O11	0.052 (3)	0.109 (4)	0.073 (4)	0.026 (3)	−0.003 (2)	−0.027 (3)
O9	0.082 (4)	0.167 (6)	0.035 (3)	0.062 (4)	0.013 (3)	0.004 (3)
O10	0.096 (5)	0.084 (4)	0.090 (4)	−0.013 (3)	0.021 (3)	−0.014 (3)
N3	0.027 (2)	0.030 (2)	0.041 (3)	−0.0025 (19)	0.006 (2)	0.002 (2)
O1W	0.071 (3)	0.041 (3)	0.106 (4)	−0.009 (3)	0.010 (3)	0.001 (2)

### Geometric parameters (Å, °)

**Table:**

Nd1—O1^i^	2.381 (3)	N4—C23	1.341 (6)
Nd1—O2	2.502 (3)	N4—C22	1.342 (6)
Nd1—O3^ii^	2.426 (3)	C22—C21	1.370 (7)
Nd1—O4^iii^	2.432 (3)	C22—H22	0.9300
Nd1—O5	2.416 (3)	C23—C24	1.366 (7)
Nd1—O7	2.406 (3)	C23—H23	0.9300
Nd1—O2W	2.492 (4)	C5—H5	0.9300
Nd1—O3W	2.564 (3)	C8—C12	1.381 (6)
Ag1—N1	2.155 (4)	C8—C7	1.521 (6)
Ag1—N2	2.155 (4)	C12—C11	1.372 (6)
Ag1—O1W^i^	2.888 (4)	C12—H12	0.9300
Ag1—O10	2.771 (5)	C14—C15	1.377 (7)
Ag2—N3^iv^	2.200 (4)	C14—C18	1.380 (6)
Ag2—N4	2.184 (4)	C14—C13	1.521 (7)
Ag2—O3W^v^	2.741 (4)	C15—C16	1.378 (7)
Ag2—O9^vi^	2.950 (5)	C15—H15	0.9300
O7—C19	1.272 (5)	C13—O6	1.232 (6)
O2—C1	1.246 (5)	C18—C17	1.372 (7)
O5—C13	1.271 (5)	C18—H18	0.9300
C3—C4	1.361 (6)	C24—H24	0.9300
C3—C2	1.387 (6)	C21—H21	0.9300
C3—H3	0.9300	C16—N3	1.348 (6)
C1—O1	1.253 (5)	C16—H16	0.9300
C1—C2	1.505 (6)	C17—N3	1.330 (7)
C2—C6	1.378 (6)	C17—H17	0.9300
N2—C11	1.348 (6)	C4—H4	0.9300
N2—C10	1.349 (6)	C11—H11	0.9300
N1—C5	1.342 (6)	C7—O4	1.241 (5)
N1—C4	1.343 (6)	C7—O3	1.241 (5)
C6—C5	1.369 (6)	O3W—H3B	0.85 (3)
C6—H6	0.9300	O3W—H3A	0.85 (3)
C10—C9	1.365 (7)	O2W—H2A	0.85 (4)
C10—H10	0.9300	O2W—H2B	0.84 (4)
C9—C8	1.381 (6)	N5—O11	1.215 (6)
C9—H9	0.9300	N5—O9	1.223 (7)
C20—C24	1.390 (6)	N5—O10	1.260 (7)
C20—C21	1.392 (7)	O1W—H1A	0.85 (3)
C20—C19	1.507 (7)	O1W—H1B	0.85 (4)
C19—O8	1.234 (6)		
			
O1^i^—Nd1—O7	81.12 (12)	O8—C19—C20	118.6 (4)
O1^i^—Nd1—O5	143.84 (12)	O7—C19—C20	115.6 (5)
O7—Nd1—O5	101.23 (13)	C23—N4—C22	117.1 (5)
O1^i^—Nd1—O3^ii^	118.20 (12)	C23—N4—Ag2	121.5 (3)
O7—Nd1—O3^ii^	139.85 (11)	C22—N4—Ag2	121.2 (4)
O5—Nd1—O3^ii^	83.25 (12)	N4—C22—C21	122.8 (5)
O1^i^—Nd1—O4^iii^	77.33 (11)	N4—C22—H22	118.6
O7—Nd1—O4^iii^	145.83 (12)	C21—C22—H22	118.6
O5—Nd1—O4^iii^	81.87 (13)	N4—C23—C24	123.2 (5)
O3^ii^—Nd1—O4^iii^	74.25 (12)	N4—C23—H23	118.4
O1^i^—Nd1—O2W	76.30 (13)	C24—C23—H23	118.4
O7—Nd1—O2W	73.28 (12)	N1—C5—C6	123.1 (4)
O5—Nd1—O2W	70.13 (12)	N1—C5—H5	118.4
O3^ii^—Nd1—O2W	142.39 (11)	C6—C5—H5	118.4
O4^iii^—Nd1—O2W	76.03 (13)	C9—C8—C12	117.4 (4)
O1^i^—Nd1—O2	71.79 (11)	C9—C8—C7	121.2 (4)
O7—Nd1—O2	76.91 (11)	C12—C8—C7	121.3 (4)
O5—Nd1—O2	144.23 (11)	C11—C12—C8	120.0 (4)
O3^ii^—Nd1—O2	76.96 (11)	C11—C12—H12	120.0
O4^iii^—Nd1—O2	119.84 (11)	C8—C12—H12	120.0
O2W—Nd1—O2	138.99 (12)	C15—C14—C18	117.7 (5)
O1^i^—Nd1—O3W	141.28 (11)	C15—C14—C13	121.5 (4)
O7—Nd1—O3W	70.39 (11)	C18—C14—C13	120.7 (5)
O5—Nd1—O3W	69.53 (11)	C14—C15—C16	120.0 (5)
O3^ii^—Nd1—O3W	74.22 (11)	C14—C15—H15	120.0
O4^iii^—Nd1—O3W	139.34 (12)	C16—C15—H15	120.0
O2W—Nd1—O3W	117.61 (12)	O6—C13—O5	126.3 (5)
O2—Nd1—O3W	76.51 (10)	O6—C13—C14	118.5 (4)
O1^i^—Nd1—H2B	75.9 (13)	O5—C13—C14	115.2 (5)
O7—Nd1—H2B	92.3 (10)	C17—C18—C14	119.1 (5)
O5—Nd1—H2B	68.0 (13)	C17—C18—H18	120.4
O3^ii^—Nd1—H2B	125.2 (6)	C14—C18—H18	120.4
O4^iii^—Nd1—H2B	56.7 (5)	C23—C24—C20	120.2 (5)
O2W—Nd1—H2B	19.3 (10)	C23—C24—H24	119.9
O2—Nd1—H2B	147.1 (13)	C20—C24—H24	119.9
O3W—Nd1—H2B	129.5 (12)	C22—C21—C20	120.3 (5)
N2—Ag1—N1	178.82 (16)	C22—C21—H21	119.9
N4—Ag2—N3^iv^	147.86 (15)	C20—C21—H21	119.9
C19—O7—Nd1	138.6 (3)	N3—C16—C15	122.2 (5)
C1—O2—Nd1	132.5 (3)	N3—C16—H16	118.9
C13—O5—Nd1	146.1 (3)	C15—C16—H16	118.9
C4—C3—C2	119.9 (4)	N3—C17—C18	123.8 (5)
C4—C3—H3	120.0	N3—C17—H17	118.1
C2—C3—H3	120.0	C18—C17—H17	118.1
O2—C1—O1	125.1 (4)	N1—C4—C3	122.5 (4)
O2—C1—C2	118.9 (4)	N1—C4—H4	118.7
O1—C1—C2	115.9 (4)	C3—C4—H4	118.7
C6—C2—C3	117.7 (4)	N2—C11—C12	122.4 (5)
C6—C2—C1	121.9 (4)	N2—C11—H11	118.8
C3—C2—C1	120.4 (4)	C12—C11—H11	118.8
C11—N2—C10	117.4 (4)	C1—O1—Nd1^vii^	163.4 (3)
C11—N2—Ag1	121.4 (3)	O4—C7—O3	126.7 (5)
C10—N2—Ag1	121.1 (3)	O4—C7—C8	116.7 (4)
C5—N1—C4	117.4 (4)	O3—C7—C8	116.5 (4)
C5—N1—Ag1	121.4 (3)	C7—O3—Nd1^iv^	135.8 (3)
C4—N1—Ag1	121.2 (3)	C7—O4—Nd1^viii^	162.1 (3)
C5—C6—C2	119.3 (4)	Nd1—O3W—H3B	122 (3)
C5—C6—H6	120.4	Nd1—O3W—H3A	108 (3)
C2—C6—H6	120.4	H3B—O3W—H3A	105.7 (17)
N2—C10—C9	122.5 (4)	Nd1—O2W—H2A	122 (4)
N2—C10—H10	118.8	Nd1—O2W—H2B	59 (4)
C9—C10—H10	118.8	H2A—O2W—H2B	106 (4)
C10—C9—C8	120.3 (4)	O11—N5—O9	122.7 (7)
C10—C9—H9	119.9	O11—N5—O10	118.7 (7)
C8—C9—H9	119.9	O9—N5—O10	118.6 (6)
C24—C20—C21	116.4 (5)	C17—N3—C16	117.0 (5)
C24—C20—C19	121.2 (5)	C17—N3—Ag2^ii^	121.6 (3)
C21—C20—C19	122.3 (4)	C16—N3—Ag2^ii^	121.4 (4)
O8—C19—O7	125.8 (5)	H1A—O1W—H1B	106 (5)

Symmetry codes: (i) *x*, −*y*+3/2, *z*−1/2; (ii) *x*−1, *y*, *z*; (iii) *x*−1, −*y*+3/2, *z*−1/2; (iv) *x*+1, *y*, *z*; (v) −*x*+1, −*y*+1, −*z*; (vi) *x*, *y*, *z*−1; (vii) *x*, −*y*+3/2, *z*+1/2; (viii) *x*+1, −*y*+3/2, *z*+1/2.

### Hydrogen-bond geometry (Å, °)

**Table:**

*D*—H···*A*	*D*—H	H···*A*	*D*···*A*	*D*—H···*A*
O1W—H1A···O10^ix^	0.85 (3)	1.96 (2)	2.796 (8)	167 (7)
O1W—H1B···O9	0.85 (4)	2.12 (5)	2.945 (8)	164 (6)
O2W—H2A···O6	0.85 (4)	2.06 (3)	2.799 (5)	145 (5)
O2W—H2B···O4^iii^	0.84 (4)	2.21 (4)	3.033 (5)	166 (5)
O3W—H3A···O8	0.85 (3)	1.87 (2)	2.653 (5)	153 (4)
O3W—H3B···O6^ix^	0.85 (3)	2.11 (3)	2.951 (5)	174 (5)

Symmetry codes: (ix) *x*, *y*, *z*+1; (iii) *x*−1, −*y*+3/2, *z*−1/2.
